# Normal Ovarian Reserve During Long-Term Osimertinib Therapy in a Patient With Li-Fraumeni Syndrome: A Case Report and Literature Review

**DOI:** 10.7759/cureus.111169

**Published:** 2026-06-19

**Authors:** Sana M Salih, Aishwarya Atmakuri, Selma Z Elsarrag, Lindsay F Schwartz

**Affiliations:** 1 Reproductive Endocrinology and Infertility, University of Chicago, Chicago, USA; 2 Obstetrics and Gynaecology, University of Chicago, Chicago, USA; 3 Internal Medicine, LewisGale Medical Center, Salem, USA; 4 Hematology Oncology, Lurie Children's Hospital, Chicago, USA

**Keywords:** epidermal growth factor receptor (egfr), fertility preservation, fumarate hydratase-deficient uterine fibroid, li-fraumeni syndrome, lung cancer, osimertinib, ovarian reserve, tyrosine kinase inhibitors (tki)

## Abstract

In contrast to the well-studied gonadotoxicity of traditional chemotherapy, data on the long-term effects of newer targeted molecular therapies on human ovarian reserves and fertility remain scarce. Here, we report the effect of osimertinib on ovarian reserve. Osimertinib is a third-generation, irreversible tyrosine kinase inhibitor that selectively targets epidermal growth factor receptor (EGFR)-mutant tumors and is used to treat EGFR-positive non-small cell lung cancer (NSCLC). We present a 30-year-old woman with a history of Li-Fraumeni syndrome (LFS), NSCLC, and a large fumarate hydratase-deficient uterine fibroid who was referred for fertility counseling and preservation while receiving maintenance osimertinib. Ovarian reserve was assessed using anti-Müllerian hormone (AMH) measurements and serial pelvic ultrasounds to determine the antral follicle count. Monitoring over 36 months of osimertinib therapy showed a normal ovarian reserve, as evidenced by normal AMH levels and a stable antral follicle count. We demonstrate a normal ovarian reserve in a patient receiving long-term osimertinib treatment. Despite a preserved ovarian reserve, patients on targeted molecular therapies for high-penetrance genetic cancers still face significant reproductive challenges. These challenges include the need for preimplantation genetic testing for monogenic disorders (PGT-M) to reduce the risk of transmitting cancer-predisposition gene mutations to their offspring and the consideration of a gestational carrier, as current guidelines prohibit pregnancy during targeted molecular therapy. Maintaining a normal ovarian reserve would allow the opportunity to delay in vitro fertilization (IVF) until a more optimal time, thereby improving reproductive planning for patients who already manage the burdens of hereditary cancer syndrome.

## Introduction

Li-Fraumeni syndrome (LFS) is a rare inherited cancer predisposition disorder caused by germline mutations in the TP53 tumor suppressor gene. LFS is an autosomal dominant disorder that confers a near 100% lifetime risk of cancer in females. These mutations lead to loss of p53 protein function and reduced ability to arrest cell cycle progression, resulting in defects in DNA repair, apoptosis, and senescence. Genomic instability caused by p53 mutations allows damaged cells to bypass checkpoints and grow uncontrollably, leading to tumor development. LFS patients have an increased incidence of lung cancer with epidermal growth factor receptor (EGFR) driver mutations. These patients are eligible for targeted molecular therapies that block EGFR signaling, which have fewer side effects than traditional chemotherapy. The two main types of targeted EGFR inhibitors are monoclonal antibodies and tyrosine kinase inhibitors (TKIs). TKIs have improved overall survival in non-small cell lung cancer (NSCLC) with EGFR-activating mutations at both early and metastatic stages [1]. First- and second-generation EGFR inhibitors, such as erlotinib, gefitinib, and afatinib, target both wild-type and mutant EGFR, whereas third-generation inhibitors, such as osimertinib, are more selective for mutant EGFR [1].

Osimertinib is a small-molecule TKI used as a first-line treatment for NSCLC harboring specific EGFR mutations. In patients with targetable EGFR-mutated advanced NSCLC, osimertinib shows up to 30-fold selectivity for mutant EGFR isoforms (T790M, L858R, and exon 19 deletion mutants) compared with the wild-type isoform [2].

An often-overlooked side effect of osimertinib is its potential impact on fertility. Osimertinib inhibits EGFR signaling, which plays multiple roles in normal ovarian function. In females, EGFR signaling influences key fertility-related processes, including ovarian follicle growth, oocyte maturation, and ovulation [3]. Although osimertinib is more selective for EGFR-mutated cancer cells than earlier-generation TKIs, it can still affect normal, healthy cells and cause side effects. In oocytes, P53 is uniquely functionally complemented by its isomer, TAp63, which helps preserve genomic integrity and provides an additional layer of protection, thereby maintaining oocyte fidelity and minimizing the effect of LFS on fertility [4]. However, data on fertility and pregnancy in patients using first- and second-generation TKIs are limited, and the impact of osimertinib on fertility remains unclear. We present a case report of a young woman with NSCLC on adjuvant osimertinib who was referred to our team for fertility preservation consultation. We show that her ovarian reserve remained normal despite prolonged osimertinib use. We discuss what is known about osimertinib's effects on fertility and pregnancy, as well as challenges and future research questions regarding reproductive health in the context of severe hereditary cancer syndromes (LFS) with autosomal dominant cancer predisposition genes (TP53).

## Case presentation

A 30-year-old Black woman, gravida 1 para 1, with a history of LFS and stage IIB NSCLC, was referred for fertility counseling. She underwent germline testing during a preconception visit because of a strong family history of cancer (her mother had two primary breast and ovarian cancers). Both she and her mother were found to carry a TP53 c.374C>A pathogenic variant, confirming LFS. Fortunately, her son tested negative. She was enrolled in LFS screening, including a whole-body MRI initiated during pregnancy. An MRI showed an indeterminate T2-hyperintense focal area in the left upper lobe of her lung measuring 2.0 x 2.1 cm. NSCLC was confirmed by transbronchial biopsy, which identified an EGFR exon 19 deletion mutation and an ataxia-telangiectasia mutated (ATM) mutation (c.2921+1G>A in ATM).

She underwent a left upper-lobe lobectomy. Her final staging was pT2aN1. Given moderate 30% PD-L1 staining, she received both adjuvant chemotherapy and targeted TKI therapy. She completed four cycles of cisplatin and pemetrexed, followed by osimertinib maintenance therapy. Because of her LFS diagnosis, she took preventive measures to reduce her future cancer risk. She underwent a simple bilateral risk-reducing mastectomy with breast reconstruction. She remained in complete remission from her NSCLC four years after her diagnosis.

**Figure 1:**
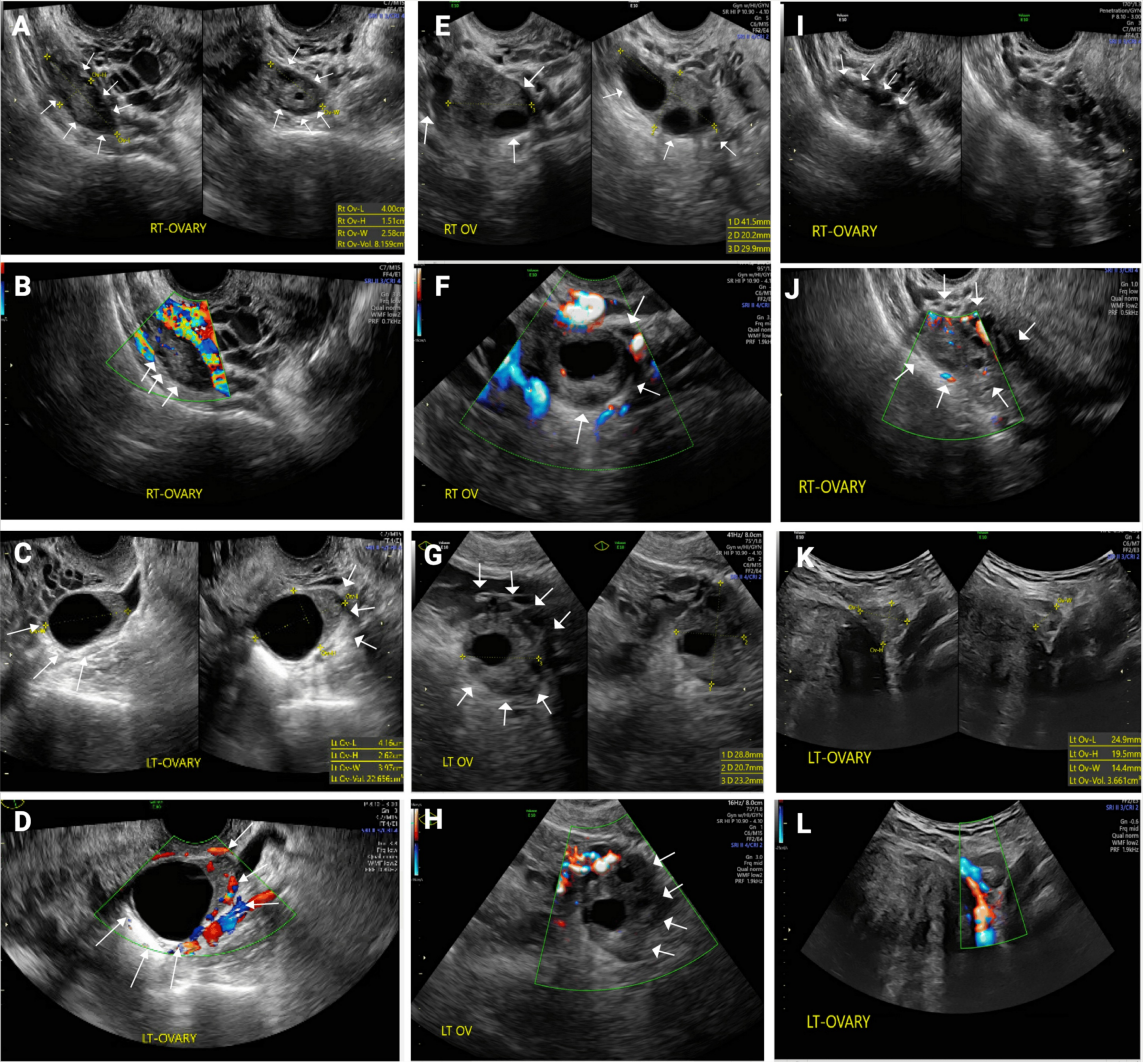
Serial annual grayscale and Doppler pelvic ultrasound scans spanning five years before the final follow-up visit A-D represent images from five years prior, obtained during a previous pregnancy, 18 months before starting osimertinib. E-H are from three years prior, and I-L are from one year prior to the final follow-up visit. A-D images were obtained during the first trimester of a previous pregnancy for comparison. A, B, E, F, I, and J represent the Rt. ovary, while C, D, G, H, K, and L represent the Lt. ovary. Ovarian images demonstrate multiple antral and growing follicles at various stages of development, indicated by well-circumscribed black circles (ovaries demarcated by white arrows). A&B, E&F, and I&J demonstrate the right ovary containing multiple antral follicles, indicating a good ovarian reserve. C&D demonstrate the left ovary containing a simple ovarian cyst, and G&H show a growing follicle in the Lt. ovary; hence, antral follicles were not visualized in these images. K & L are transabdominal ultrasounds, with L showing increased Doppler flow indicative of increased pelvic vascularity around the left ovary. Scale bars represent approximately 1 mm.

**Figure 2:**
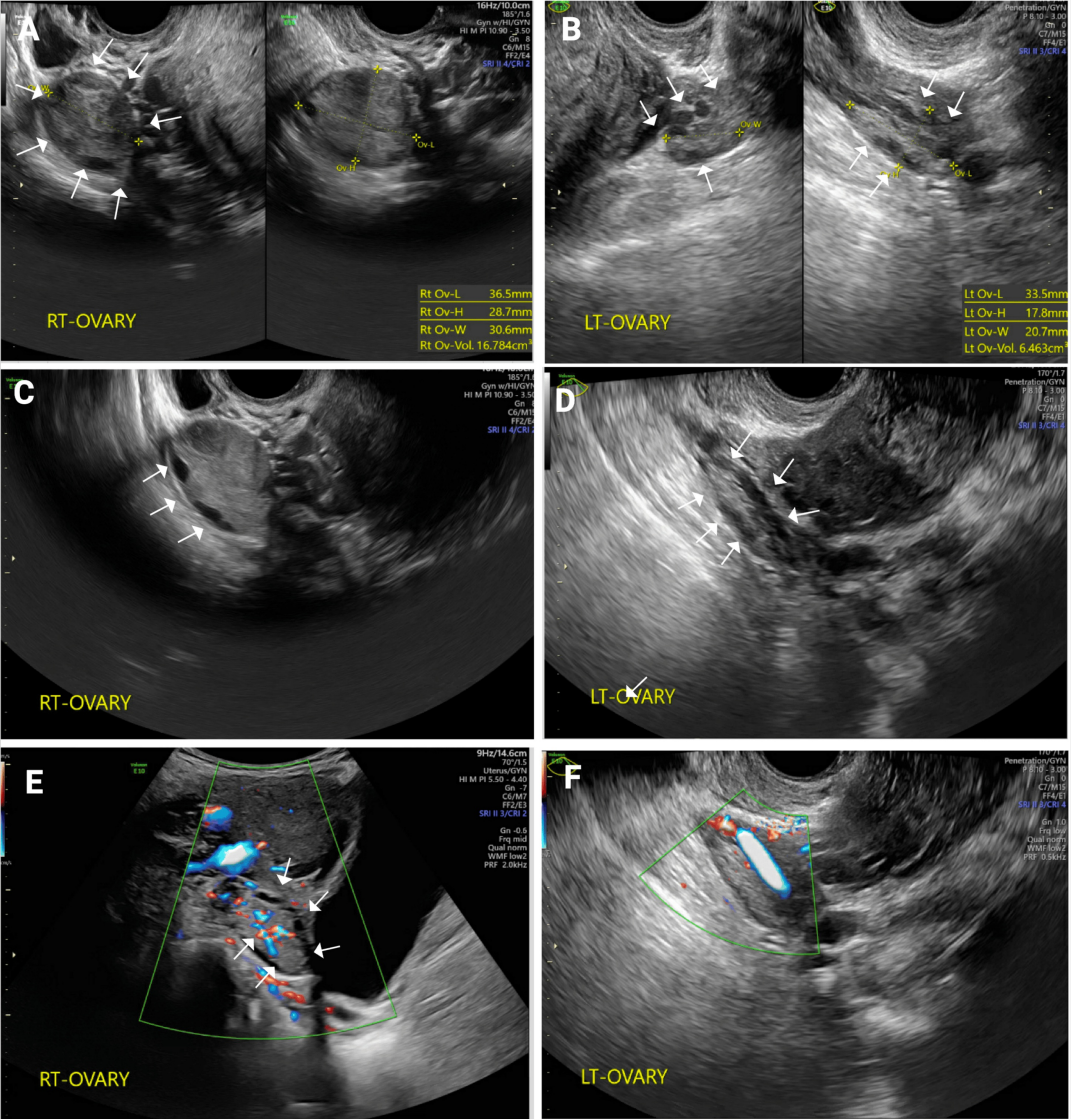
Grayscale and Doppler pelvic ultrasound images obtained three months before the final follow-up visit A, C, and E represent the Rt. ovary, while B, D, and F represent the Lt. ovary. A-D show a good number of antral follicles in both ovaries, indicated by well-circumscribed small black circles, consistent with normal ovarian reserve (ovaries demarcated by white arrows). E and F show Doppler flow with increased vascularity around both ovaries, including a large blood vessel adjacent to the Lt. ovary that could complicate vaginal access to the left ovary. E is a transabdominal image of the right ovary, demonstrating that it is displaced upward by the fibroid and located higher in the pelvis. Scale bars represent approximately 1 mm.

The patient was counseled on fertility preservation with embryo cryopreservation and preimplantation genetic diagnosis for TP53 mutations due to concerns about passing LFS to her children and uncertainty about the effects of osimertinib on fertility. The patient underwent serial pelvic ultrasounds to assess her ovarian reserve. Transvaginal ultrasounds showed a good ovarian reserve, with an antral follicle count of about 40, which remained stable despite 36 months of osimertinib therapy (Figures 1-2). Similarly, her AMH level was 4.4 ng/mL (normal: 1-5 ng/mL); the repeat AMH was 3.7 ng/mL, still within the normal range, despite ongoing osimertinib therapy. Serial pelvic ultrasound images of the ovaries obtained over a five-year period showed a stable antral follicle count and an enlarging uterine fibroid (Figure 1). Ovarian follicles appear as small, rounded, black circles within the ovary, representing the fluid-filled antrum of antral and growing follicles. Ovarian follicles at various stages of growth were observed in both ovaries. Only follicles with a mean diameter of 2-10 mm are counted as true antral follicles. Serial images obtained 18 months before osimertinib therapy, compared with those obtained over a three-year period during osimertinib therapy, show that both ovaries contain multiple antral follicles, indicating a maintained ovarian reserve while on osimertinib therapy (best seen in Figures 1A, 1B, 1I, 1J). Repeat imaging three months before the final follow-up visit showed that her antral follicle count remained stable, with multiple antral follicles in both ovaries (Figure 2).

**Figure 3:**
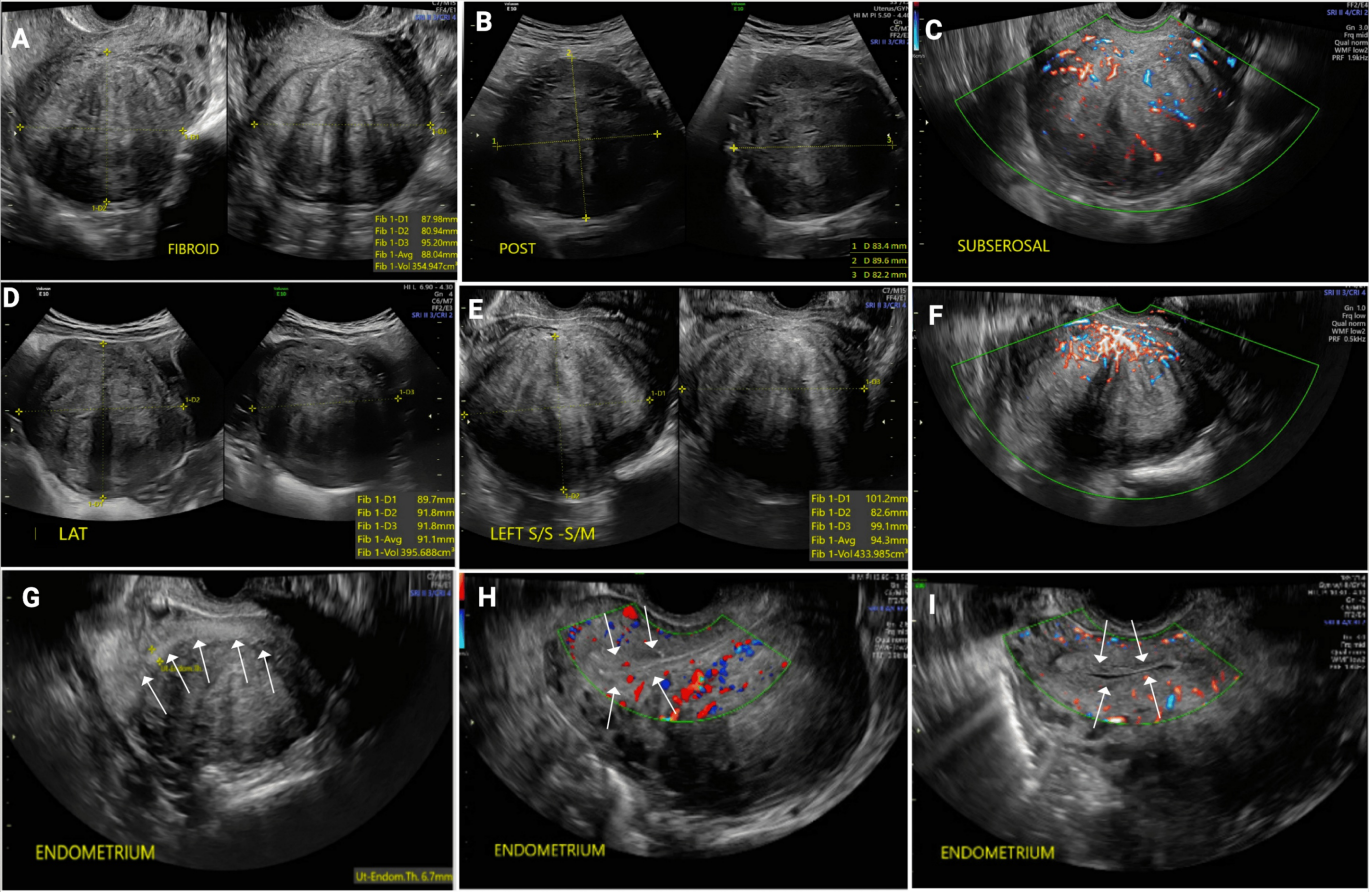
Serial annual pelvic ultrasound scans (grayscale and Doppler) performed over the five years preceding the final follow-up visit A-D represent images from five, four, three, and one years prior, and E-G represent images from three months before the final follow-up visit. A-E show the uterine fibroid as a hypoechoic mass with acoustic shadowing. C and F show positive Doppler flow, consistent with increased internal and peripheral blood flow within the fibroid. G shows the fibroid impinging on the endometrium and displacing it anteriorly (arrows). H and I are images obtained three years before the final follow-up visit; H shows a normal trilaminar endometrial lining, while I shows an anechoic intracavitary fluid accumulation within the endometrial cavity (arrows). Scale bars represent approximately 1 mm.

The patient experienced menorrhagia, leading to iron-deficiency anemia caused by a solitary International Federation of Gynecology and Obstetrics (FIGO) 2-5 intramural uterine fibroid, measuring an average of 9.4 cm in diameter. Serial pelvic ultrasound images of the fibroid obtained over a five-year period before the final follow-up visit showed an uneven, heterogeneous echotexture with mixed internal echoes (Figure 3). The fibroid increased in size by 20% over five years but remained similar in appearance and exhibited positive Doppler flow, consistent with increased internal and peripheral blood flow signals within the fibroid and around both ovaries. The fibroid displaced the right ovary upward, limiting access to the ovary for transvaginal oocyte retrieval and increasing the risk of bleeding. Although the endometrial cavity appeared normal with trilaminar echoes, the fibroid impinged on it, displacing it anteriorly, and fluid accumulated within the cavity. The distortion of the endometrial lining and the accumulation of endometrial fluid were attributed to the fibroid and could reduce fertility by preventing embryo implantation. 

Our team recommended myomectomy before fertility preservation to facilitate access to the ovaries for future oocyte retrieval. She underwent an uneventful robot-assisted laparoscopic myomectomy. Pathology of the fibroid tumor showed hemangiopericytoma-like vessels and bizarrely nucleated areas, and molecular fingerprinting confirmed a fumarate hydratase (FH)-deficient leiomyoma, indicating a high risk of recurrent hereditary leiomyoma. We discussed in vitro fertilization with prenatal genetic testing for monogenic disorders (IVF with preimplantation genetic testing for monogenic disorders (PGT-M)) to reduce the risk of LFS transmission to her offspring and to consider a gestational carrier to avoid withholding cancer therapy for prolonged periods. Although reassured by a normal ovarian reserve, she was overwhelmed by the prospect of IVF during her many other treatment fronts and the need to consider a gestational carrier. She decided against immediate IVF and genetic testing of embryos for TP53, opting instead to try later when and if she can safely discontinue osimertinib and become pregnant.

## Discussion

Challenges of fertility preservation and LFS

Most cancer patients of reproductive age worry about how treatment might affect their fertility and what preservation options are available. Planning ovarian stimulation for fertility preservation in cancer patients is often challenging because of timing constraints, coexisting health conditions, and the risk of cancer recurrence if cancer therapy is interrupted. This case report contributes to the limited literature on potential safety and toxicity concerns associated with targeted molecular cancer therapy, such as osimertinib, in the context of fertility and fertility preservation. We report that the ovarian reserve remained normal while the patient was taking osimertinib and acknowledge the challenges of fertility planning and coordination related to osimertinib and TKI therapy.

The patient's AFC and AMH remained within the normal range throughout the observation period despite osimertinib and chemotherapy. Cisplatin is moderately toxic, and its ovarian effects depend on the patient's age, ovarian reserve, and cumulative dose. True ovarian reserve refers to the number of primordial follicles in the ovary, which cannot be measured directly because of their small size and dormancy. In laboratory animal studies, serial sectioning of the ovaries with H&E staining and follicle counting are used to precisely assess ovarian reserve. In humans, a few surrogate markers of ovarian reserve exist, with AMH and AFC emerging as the best predictors. AMH and AFC are interpreted using range groups rather than specific numbers. AFC varies slightly across menstrual cycles, depending on cycle day, hormone treatments, and the presence or absence of dominant follicles. However, the AFC range usually remains consistent within a given patient across many cycles. AMH is secreted by granulosa cells of preantral and small antral follicles and serves as a proxy for the number of follicles in the pipeline. Common range groups accepted in reproductive medicine include very low ovarian reserve (AMH < 0.5, AFC 0-4); low ovarian reserve (AMH 0.5-1.2, AFC 5-9); normal ovarian reserve (AMH > 1.2-4.5, AFC 10-20); and high ovarian reserve (AMH > 4.5, AFC > 20) [5]. It is important to note that ovarian reserve does not correlate with natural conception. Women with regular menstrual cycles would have age-matched fertility potential regardless of their ovarian reserve, since only one mature egg is needed for natural conception. Ovarian reserve, however, is essential for predicting response to ovarian stimulation medications and the total number of eggs retrieved during an IVF cycle, since the latter depends on the growing follicle pool.

Given the profound gap in reproductive health regarding the long-term integrity of oocytes in patients with P53 genomic instability and LFS, and in those exposed to osimertinib therapy, fertility and fertility preservation are not just a quality-of-life consideration but an essential step. Maintaining a normal ovarian reserve reduces the urgency of fertility preservation, allowing the patient to consider it when cancer is better controlled, while carefully weighing other factors, such as age-related fertility decline, the unknown risk of cancer recurrence, and the possibility of needing gonadotoxic cancer therapy, thereby supporting better reproductive planning.

In addition to fertility, she was counseled about the need for IVF with PGT-M to decrease the risk of transmitting the cancer-predisposition gene mutation (TP53) to her offspring. Patients with the rare LFS diagnosis who wish to preserve fertility face unique genetic challenges. The TP53 germline mutation that causes LFS is an autosomal dominant condition with a 50% chance of transmission to each child. PGT-M of embryos can help reduce this risk once the family's specific mutation is identified. IVF with PGT-M for monogenic disorders is considered the standard medical recommendation for a patient wishing to prevent passing a severe, dominant cancer predisposition gene, such as TP53 and LFS, to their offspring. However, recommending IVF with PGT-M creates complex clinical and logistical challenges and necessitates substantial social and emotional support to address financial and socioeconomic barriers while respecting patient autonomy. Patients with LFS might need to consider third-party reproductive options, such as a gestational carrier, if pausing TKI therapy during pregnancy is unsafe. Pregnancy may add risks, as some studies have shown that a germline TP53 mutation can be transmitted from a male LFS carrier to the embryo of a female partner, increasing the risk of choriocarcinoma and other gestational trophoblastic diseases [6].

Impact of osimertinib therapy on fertility

The EGFR pathway is crucial for the proper function of reproductive tissues. In the female reproductive system, EGFR signaling relays the effects of LH, FSH, and growth factors from granulosa cells to the oocyte, thereby supporting antral follicle growth, differentiation, oocyte maturation, cumulus expansion, and ovulation [3]. EGFR signaling primarily affects growing follicles and oocyte maturation rather than primordial follicles, which constitute the ovarian reserve. Consequently, unlike traditional chemotherapy, which often destroys primordial follicles and reduces the ovarian reserve, EGFR inhibitors, such as TKIs, primarily target growing follicles. If primordial follicles remain unaffected, fertility could potentially recover after discontinuing TKIs.

Although osimertinib is more selective for EGFR-mutated cancer cells than for wild-type EGFR, it can still affect healthy cells and cause side effects [1]. Osimertinib has been shown to temporarily impair female fertility in rats, causing anestrus, degeneration of the corpus luteum (consistent with the role of EGFR in ovulation), and thinning of the epithelium in the uterus and vagina [7]. Initial pharmaceutical studies showed that rat fertility returned one month after cessation of osimertinib treatment. This aligns with osimertinib's impact on EGFR signaling, which is crucial for antral follicle growth, particularly oocyte maturation, cumulus expansion, and ovulation, rather than for primordial follicle development and maintenance. It also matches our patient's case, in which AMH levels and ovarian reserve remained normal despite three years of osimertinib treatment.

A third-generation TKI such as osimertinib, which specifically targets the mutated form of the EGFR gene, is likely to have limited off-target effects due to minimal cross-reactivity with normal EGFR [2]. It is possible that this will thereby minimize harm to the normal ovarian reserve. However, clinical studies confirming this in reproductive tissue are lacking. Current clinical guidelines recommend that female patients use contraception for at least six to eight weeks after the final dose of osimertinib before trying to get pregnant, to allow the drug to be cleared from the body. Additionally, clinical guidelines prohibit pregnancy while on osimertinib therapy, as animal studies have indicated it can cause fetal harm [7]. For many patients with EGFR-mutant lung cancer, stopping osimertinib therapy during IVF stimulation and pregnancy may pose a significant risk of cancer progression, as up to 23% of patients with EGFR-mutant lung cancer who discontinued TKI experienced a disease flare eight days after drug interruption [8]. The limited ability of patients to interrupt osimertinib to become pregnant while taking osimertinib requires the use of IVF and a gestational carrier.

The duration of osimertinib treatment is based on cancer stage, treatment intent to prevent recurrence or to manage active disease, and recurrence status [9]. A key consideration is how long to pause long-term adjuvant cancer therapy before IVF. Because human studies are unavailable, the effects of osimertinib on oocyte quality and integrity, as well as on long-term ovarian reserve, remain unknown. Based on limited medical literature, we cautiously recommend waiting two weeks before starting ovarian stimulation for fertility preservation and a total of four weeks before oocyte retrieval. Osimertinib has a short half-life of about 48 hours, and it typically takes around 10 days for the drug to be eliminated from the body. Pharmacokinetic data indicate that total clearance from the bloodstream takes roughly 10 days (five half-lives, 240 hours) after the last dose. Therefore, we recommend waiting four weeks before egg retrieval for fertility preservation, although shorter or longer wait periods may be reasonable based on future studies. Beyond ovarian reserve and oocyte number, future human studies are needed to carefully assess the effects of osimertinib and TKIs on oocyte quality, fidelity, and competence; embryo development; pregnancy outcomes; and the transgenerational impact of these agents. These studies would identify any detrimental effects of osimertinib on growing oocytes and inform whether shorter or longer wait periods may be reasonable before oocyte retrieval.

TKI and fertility preservation

There is no clinical data on the effects of osimertinib and other TKIs on fertility or fertility preservation in humans, but two case reports describe successful IVF with ovarian stimulation in patients taking crizotinib. Oocyte retrieval was performed safely, resulting in healthy babies without disease progression, even after brief discontinuation of medication. Gillis et al. briefly described the first case of a patient who underwent two autologous IVF cycles while on crizotinib. Two embryos were transferred to a gestational carrier, and the couple successfully had healthy dizygotic twins [10]. Su et al. reported on a 36-year-old woman who underwent two IVF cycles after stopping crizotinib [11]. She had a normal ovarian reserve, with an AMH of 3.7 ng/mL and a normal antral follicle count, despite being on treatment for three years. Crizotinib has a 42-hour half-life. It was discontinued 16 days before ovarian stimulation, for a total of 31 days off, and was restarted on the day of oocyte retrieval without any disease progression. The couple successfully achieved live births of dizygotic twins via gestational surrogacy [11]. These cases demonstrate that the ovaries can recover quickly and respond to stimulation after TKI withdrawal [11]. However, they also highlight a preference for transferring two embryos to the gestational carrier to allow the intended parents to complete their family in one round of gestational carrier, which is contrary to current recommendations for single embryo transfer and underestimates the increased risk of fetal and maternal complications associated with multiple gestations. In the case of crizotinib, the FDA label recommends that females use effective contraception for at least 90 days after the final dose of crizotinib to ensure a complete "washout" of germ cells (oocytes) that might have been affected. Still, these cases show that a much shorter interruption period is possible and compatible with having healthy children [11]. The authors discussed ethical issues related to biological therapies, such as the debate over the right to parenthood while managing a progressive, incurable disease with a potentially limited life expectancy, and the right to pursue reproductive treatments while on such therapies [10]. These two case reports emphasize the challenges of fertility preservation during long-term treatment with targeted molecular therapies. They also highlight the limited knowledge of how these therapies impact oocytes, embryos, and pregnancy, as well as interruptions to cancer treatments to support fertility.

Impact of other TKIs on fertility

Evidence on how other TKIs affect fertility in animal models is limited, and it remains unclear how much can be inferred about osimertinib, given the distinct roles of growth signaling pathways in the female reproductive system [7]. The specific effects of TKIs on fertility are not fully understood, as evidence indicates both protective and harmful effects on the ovary, highlighting a gap between the biological roles of kinases and their effects on fertility [12]. Animal studies suggest that afatinib, gefitinib, and imatinib may reduce fertility, whereas lapatinib, sunitinib, and everolimus do not, highlighting that each targeted therapy is distinct and requires separate evaluation [12-15]. Human studies showed that lapatinib and trastuzumab did not increase the rate of amenorrhea in premenopausal women with breast cancer when administered without chemotherapy [16]. Gefitinib, on the other hand, was reported to reduce androgen levels in both men and women and to decrease sperm count and motility [17]. This complexity is compounded by crosstalk between EGFR and other signaling pathways that may compensate for EGFR inhibition. One study reported that lapatinib had minimal effects on ovarian reserve and reproductive function in a mouse model, likely due to activation of the STAT3 signaling pathway, which could counteract lapatinib's inhibition of EGF receptors and help maintain ovarian function [15]. Therefore, further research is necessary. These findings cannot be directly applied to humans because of differences in reproductive lifespans and the polyovulatory nature of rodents, including short estrous cycles, the absence of menstruation, and distinct ovarian aging mechanisms, underscoring the need for more human data.

Impact of osimertinib on pregnancy and ethics

Using TKIs during pregnancy poses unique challenges due to potential fetal risk and the ethical considerations of pursuing parenthood while managing a progressive disease [7]. Animal data from the drug's pharmaceutical approval process showed that osimertinib adversely affected embryonic and fetal development in pregnant rats, increasing post-implantation loss and early embryonic death. The manufacturer recommended continuing contraception during treatment with osimertinib and for six weeks for females and four months for males after stopping osimertinib, and advised against breastfeeding for two weeks after stopping osimertinib. Two case reports described pregnancies during osimertinib treatment in women with EGFR-mutant NSCLC that were complicated by oligohydramnios and intrauterine growth restriction, yet two healthy babies were born [18,19]. One patient was on afatinib, which was discontinued before six weeks of gestation after pregnancy was documented. To prevent cancer recurrence, the patient started osimertinib in the second trimester and delivered at 37.3 weeks. The second patient was diagnosed with NSLC and brain metastases at 23 weeks' gestation. She underwent resection of the brain lesions and received osimertinib at 27 weeks, followed by trastuzumab a week later. She had spontaneous preterm labor at 30 weeks. Both pregnancies were complicated by intrauterine growth restriction and severe oligohydramnios. Both babies had no congenital anomalies, and one developed transient renal failure that resolved at 17 days of life. These two cases highlight that osimertinib may be used to manage tumors during pregnancy, but it does not replace current guidelines on avoiding pregnancy and using contraception [18]. While official guidelines still advise avoiding pregnancy and using contraception during osimertinib treatment, these two cases demonstrate that osimertinib can successfully manage EGFR-mutant lung cancer during pregnancy, as both cases resulted in healthy babies, though both were complicated by low amniotic fluid and restricted fetal growth.

LFS and uterine fibroids

Multiple hereditary and genetic factors intersect to complicate reproductive planning for patients with LFS. While LFS does not typically increase the risk of benign tumors, it has been associated with mutations in the FH gene and with an increased risk of FH-negative fibroids that is independent of P53 mutations. Indeed, the pathology report of the fibroid revealed FH-deficient uterine leiomyomas, a distinct, often hypercellular, atypical subtype of uterine fibroids. FH is a vital metabolic enzyme in the Krebs cycle and a tumor suppressor oncometabolite. A mutation in the FH gene leads to excess fumarate and inhibition of ten-eleven translocation (TET) hydroxylases, which maintain normal DNA methylation patterns. When TET is blocked, cellular machinery is geared to promote fibroid tumor growth [20]. FH-deficient uterine fibroids can increase the risk of infertility and early pregnancy loss due to the hyper-vascularized and distorted uterine environment. These fibroids are also associated with an increased risk of early hysterectomies because of the tumor's recurrent and aggressive nature. Large fibroids associated with LFS can increase complications during the IVF cycle by blocking vaginal access to the ovaries and increasing pelvic blood flow, making transvaginal oocyte retrieval more difficult. Although transabdominal retrieval is an option, it is rarely performed. Additionally, fibroids may cause fluid buildup in the endometrial cavity, leading to infertility due to failure of embryo implantation. FH-deficient leiomyomas result from somatic or germline mutations in the FH gene. Germline mutations in the FH gene occur in a minority of FH-deficient uterine fibroids and are associated with an increased risk of uterine, cutaneous, and renal leiomyomas, as well as renal cell carcinoma. These patients can benefit from preimplantation genetic diagnosis of embryos to reduce the risk of passing on the gene mutation to offspring.

## Conclusions

Integrating targeted molecular cancer therapies, such as osimertinib, into first-line protocols for reproductive-aged patients requires a shift in fertility counseling and oncofertility research. This compelling case report addresses an important gap in clinical knowledge by examining the intersection of a targeted oncological therapy (osimertinib), a hereditary cancer predisposition syndrome (LFS), and reproductive health (ovarian reserve and uterine fibroids). Because traditional chemotherapies are markedly gonadotoxic, documenting a normal ovarian reserve after long-term TKI use provides invaluable data for the growing field of oncofertility. A limitation of this report is that the patient has declined fertility preservation with PGT/M and a gestational carrier. As such, we do not know the effects of pausing osimertinib on embryo quality and pregnancy outcomes, highlighting a significant knowledge gap. While our case shows that ovarian reserve appears to remain normal with prolonged osimertinib exposure, a careful balance between retrieving high-quality oocytes and the risk of cancer progression warrants careful consideration. Furthermore, the ethics of attempting natural conception without PGT/M, despite a known strong genetic cancer predisposition risk for offspring, calls for a delicate balance between prevention and supportive care. This case highlights the knowledge gap regarding fertility and pregnancy outcomes during TKI therapy. It calls for establishing prospective multicenter registries and standardized fertility surveillance protocols to conduct long-term follow-up studies of the reproductive health of children, women, and men receiving targeted cancer therapies and to develop evidence-based reproductive standards for these patients.
